# Obituary

**Published:** 1895-04

**Authors:** 


### Obituary.

DR. DUJARDIN-BEAUMETZ, editor-in-chief of the *Bulletin Général de Thérapeutique*, died in Paris, February 16, 1895. His death was somewhat sudden and he left much literary work uncompleted. He has often been quoted as authority on therapeutic subjects. He was preparing an article on diseases of the muscles, for the *Twentieth Century Practice of Medicine*, now publishing by Messrs. Wm. Wood & Co.

DR. DANIEL HACK TUKE, the eminent alienist and author of works on mental disease, died in London, March 6, 1895, aged 68 years.

